# First year-round occurrence checklist of mesopelagic “swimmers” collected by an automatic sediment trap on Mooring B (Ross Sea, Antarctica) in years 1995-1996, 2004-2005 and 2014-2016 

**DOI:** 10.3897/BDJ.14.e197009

**Published:** 2026-07-29

**Authors:** Marco Grillo, Martina Linciano, Maria Balan, Alice Guzzi, Nicholas Francesco Noli, Valentina Cometti, Leonardo Langone, Patrizia Giordano, Stefano Schiaparelli

**Affiliations:** 1 Italian National Antarctic Museum (MNA, section of Genoa), Genoa, Italy Italian National Antarctic Museum (MNA, section of Genoa) Genoa Italy; 2 Department of Earth, Environmental and Life Sciences (DISTAV), Genoa, Italy Department of Earth, Environmental and Life Sciences (DISTAV) Genoa Italy; 3 National Biodiversity Future Center (NBFC), Palermo, Italy National Biodiversity Future Center (NBFC) Palermo Italy; 4 Department of Physical Sciences, Earth and Environmental (DSFTA), Siena, Italy Department of Physical Sciences, Earth and Environmental (DSFTA) Siena Italy; 5 CNR-ISP – National Research Council – Institute of Polar Sciences, Bologna, Italy CNR-ISP – National Research Council – Institute of Polar Sciences Bologna Italy

**Keywords:** Antarctic zooplankton, Antarctic biodiversity, pelagic community, time-series analysis

## Abstract

**Background:**

Long-term studies of pelagic communities in the Southern Ocean are crucial for understanding biodiversity patterns and detecting the impacts of climate change and other environmental stressors. Here, we provide temporal occurrence and spatial data on Antarctic “swimmers” (i.e. pelagic organisms that are captured by sediment traps deployed on moorings) collected within three projects of the Italian National Antarctic Programme (PNRA) in the Ross Sea sector during the periods 1995–1996, 2004–2005 and 2014–2016. These data were obtained over six Italian Antarctic expeditions carried out between 1994 and 2016.

**New information:**

This dataset constitute to the first checklist of Antarctic “swimmers” collected in the Joides Basin, a sector of the Ross Sea (Antarctica), using automatic sediment traps deployed at approximately 500 metres depth on Mooring B (approx. Lat = 74.1514°S, Lon = 175.0906°E). Although primarily designed to study particle and export fluxes, these traps also capture motile pelagic organisms, the “swimmers”, that enter actively or accidentally. In total 210,411 individuals were sampled. These represent 68 genera and 68 species, corresponding to 905 occurrence records. These accidental captures provide valuable biodiversity data for zooplankton and micronekton taxa yielding quality information useful to refine species distributional models and occurrence predictions, due to the fact that, together with the biological records, a full set of environmental data (e.g. temperature, salinity and density) continuously collected by mooring sensors.

## Introduction

### The "swimmers" of the Joides Basin (Ross Sea): a window on Antarctic mesopelagic biodiversity

Antarctic marine ecosystems play a pivotal role in regulating the global climate and in the biogeochemical cycles of the ocean (Henley et al. 2020, Constable et al. 2023, Schofield et al. 2024). In particular, the Ross Sea represents one of the most productive and dynamic areas of the Southern Ocean, hosting planktonic and benthic communities that respond sensitively to environmental changes (Smith et al. 2014, Cummings et al. 2018, D'Sa et al. 2021, Kim et al. 2022, Thomalla et al. 2023). Amongst the communities inhabiting the Ross Sea, “swimmers” play a crucial role in carbon cycling through trophic transfer and vertical transport of organic matter (Atherden et al. 2024). The term “swimmers” refers to all mobile marine organisms that, due to their swimming capabilities, whether in adult phase or larval stages, enter, either actively or accidentally, into structures or devices designed for other purposes, such as sediment traps (Langone et al. 2000, Langone et al. 2003, Chiarini et al. 2013, Chiarini et al. 2019, Atherden et al. 2024, D'Angelo et al. 2024).

Their presence is not solely the result of passive transport; they can also move autonomously, actively reaching the trap cup, where they are subsequently collected and preserved in the formalin solution (Knauer et al. 1979, Matsuno et al. 2014, Makabe et al. 2016, Danovaro et al. 2017). In the first case, the organisms reach the jar already dead, while, in the second case, organisms are perfect and were killed by the preservative solution.

The swimmers category is mainly composed of zooplanktonic, micronektonic and benthic organisms and primarily include planktonic crustaceans, such as copepods, euphausiids and amphipods, as well as tunicates (appendicularians and salps), cnidarians, pelagic molluscs (pteropods), chaetognaths and larval stages of fish and decapod larvae (Atherden et al. 2024, D'Angelo et al. 2024).

Zooplanktonic taxa are a fundamental component of marine ecosystems, acting as a key link between primary producers (e.g. phytoplankton community) and higher trophic levels, such as fish, bird and marine mammals (Granata et al. 2000, La Mesa et al. 2000, Turner 2004, Pakhomov et al. 2020). Additionally, some zooplanktonic groups actively contribute to the vertical transport of carbon through the production of faecal pellets and daily migrations within the water column (Atkinson 1996, Hansen et al. 1997, Carli et al. 2000, Calbet and Landry 2004, Ratnarajah et al. 2023).

Zooplankton respond sensitively to environmental changes (e.g. water column stratification and ocean acidification) (Barton et al. 2013), with effects on their abundance, distribution, life cycle and physiological adaptations as highlighted in recent studies (Poloczanska et al. 2013, Kim et al. 2022). Zooplankton assemblages in particular and, especially copepods, represent a reliable indicator (Edwards and Richardson 2004, Hays et al. 2005, Edwards 2009) of environmental variability, including both natural and human-induced climate change perturbations (Turner 2004, Bonello et al. 2020).

Zooplankton communities in the Ross Sea sector have been extensively studied since 1985 with a special focus on their biodiversity and ecological role (Amato 1990, Carli et al. 1990, Guglielmo et al. 1990, Zunini Sertorio et al. 1990, Carli et al. 1992, Zunini Sertorio et al. 1994, Acosta Pomar et al. 2000, Carli et al. 2000, Zunini Sertorio et al. 2000, Carli et al. 2002,Pane 2004, Bolinesi et al. 2020, Cecchetto et al. 2021, Cecchetto et al. 2022, Cordone et al. 2022, Grillo et al. 2022, Monti-Birkenmeier et al. 2022, Barra et al. 2023, Bergamasco et al. 2023, Grillo et al. 2024b, Minutoli et al. 2025) and their relationship with sea ice (Guglielmo et al. 2007, Granata et al. 2009, Guglielmo et al. 2015, Granata et al. 2022). The micronectonic and benthic communities have also been extensively studied, as they play an ecological role and have been the subject of numerous studies aimed at understanding their biodiversity and ecological dynamics in the Ross Sea (Azzali and Kalinowski 2000, Faranda et al. 2000, Vacchi et al. 2000, Schiaparelli et al. 2006, Piazza et al. 2020).

Here, we present a complete checklist of “swimmers” collected by automatic sediment traps during six different Italian Antarctic expeditions, from '1995' to '2016', all placed on Mooring “B” in the Joides Basin. The positioning of the sediment trap(s) corresponded to the mesopelagic layer, at depths between 470 and 530 metres, a still undersampled ecological zone and may provide relevant information for modelling species distribution (Grillo et al. 2022, Grillo et al. 2024a, Grillo et al. 2024b).

These samples are now curated by the Italian National Antarctic Museum (MNA, section of Genoa), whose previous contributions focused on Mollusca, Tanaidacea, Fungi, Ophiuroidea, Porifera, Bryozoa, Rotifera, Asteroidea, Copepoda, Isopoda and benthic pionieer communities (Ghiglione et al. 2013, Piazza et al. 2014, Selbmann et al. 2015, Cecchetto et al. 2017, Ghiglione et al. 2018, Cecchetto et al. 2019, Bonello et al. 2020, Garlaschè et al. 2020, Guzzi et al. 2022, Grillo et al. 2024c, Noli et al. 2024, Cometti et al. 2025).

## Project description

### Title

First year-round occurrence checklist of mesopelagic organisms (“swimmers”) collected by an automatic sediment trap on Mooring B (Ross Sea, Antarctica) in years 1995-1996, 2004-2005 and 2014-2016

### Personnel

Grillo Marco, Linciano Martina, Balan Maria, Guzzi Alice, Noli Nicholas, Cometti Valentina, Langone Leonardo, Giordano Patrizia, Schiaparelli Stefano.

### Study area description

Ross Sea area, being a high-latitude, nutrient-rich continental shelf system, supports extensive phytoplankton blooms each spring, driven by the seasonal retreat of sea ice and the recurrent formation of coastal polynyas, particularly near the Ross Ice Shelf and Terra Nova Bay (Langone et al. 2003, Burgay et al. 2020, Fernandez et al. 2025). These oceanographic and ecological dynamics directly affect the Joides Basin, integrating it into the wider biological productivity patterns of the region, as documented in early studies (Smith and Nelson 1985, Nelson and Smith 1986, Russo 2000). Joides Basin, located in the western Ross Sea (Fig. 1), is a prominent shelf basin elongated in a southwest–northeast direction. It forms part of the broader, deep epicontinental shelf that characterises the Ross Sea region. As a shelf basin, Joides acts as a sediment trap, accumulating materials transported from surrounding glacial margins and the overlying water column (Corradi et al. 1997, Langone et al. 2000, Torricella et al. 2021). Samples were collected in the Joides Basin area through the deployment of Mooring B, which was equipped with an automated sediment trap positioned at different depths depending on the sampling year (ranging from 470 and 530 mteres). The seafloor at the site lies at approximately 560 metres. Sampling campaigns were carried out during three different Italian Antarctic Expeditions: 1994–1995, 2004–2005 and 2014–2016. Each sampling time series spanned two consecutive expeditions, to allow for mooring deployment in the first year and recovery in the following one. During these campaigns, the sediment trap was deployed at a depth of 530 metres in 1994–1995, 515 metres in 2004–2005 and 470 metres in 2014–2016. Mooring B was consistently deployed within the Ross Sea Marine Protected Area (MPA), specifically in sector RS-GPZi (Ross Sea - General Protection Zone), for all time series.

In 2009, the long-term monitoring programme MORSea – Marine Observatory in the Ross Sea was launched, coordinated by the University of Naples “Parthenope” in collaboration with the National Research Council (CNR). The project is dedicated to managing the numerous oceanographic moorings deployed in the western sector of the Ross Sea since 1994. The main objective is to investigate the physical and biogeochemical characteristics of the water mass located above the continental shelf of the Ross Sea, an area where dense shelf waters are formed. To achieve this, the programme collects oceanographic data and develops time series that allow researchers to monitor environmental changes over time (Project Mooring Page).

### Funding

Data originated in the framework of the PNRA Antarctic Italian Expeditions carried out in several research project funded by the PNRA:

- X Italian expedition (1994/1995), Project: ”Biogenic sedimentation". Project code PEA94; Scientific coordinator: Dr. Ravaioli Mariangela;

- XI Italian expedition (1995/1996), Project: ”Biogenic sedimentation". Project code PEA94; Scientific coordinator: Dr. Ravaioli Mariangela;

- XIX Italian expedition (2003/2004), Project: ”BIOSESO". Project code PEA2003-04; Scientific coordinator: Dr. Ravaioli Mariangela;

- XX Italian expedition (2004/2005), Project: ”BIOSESO". Project code PEA2003-04; Scientific coordinator: Dr. Ravaioli Mariangela;

- XXIX Italian expedition (2013/2014), Project: ”RoME/UR6-Biogeochemistry”. Project code PNRA2013/AN2.04/UR6; Scientific coordinator: Dr. Langone Leonardo;

- XXXI Italian expedition (2015/2016), Project: ”RoME/UR6-Biogeochemistry”. Project code PNRA2013/AN2.04/UR6; Scientific coordinator: Dr. Langone Leonardo;

## Sampling methods

### Sampling description

The sediment trap was designed to capture sinking particulate matter over the course of the deployment period, enabling the collection of biological material descending through the water column.

For the 1995–1996 time series, a McLane Parflux Mark 7G-21 cups trap with a 0.5 m² collection area was used. For the 2004–2005 and 2014–2016 time series, a McLane 78HW-13 cups trap, also with a 0.5 m² collection area, was deployed. The cups were previously filled with a buffered solution of 5% formaldehyde to better preserve the organic matter and the cup rotation intervals varied between 7 days to 3 months.

More information on the technical specifications of Mooring B and the pre-treatment of samples can be found in Langone et al. (2000) and Langone et al. (2003).

### Quality control

All records were validated. Coordinates were converted into decimal latitude and decimal longitude and plotted to verify the geographical location and locality. All scientific names were checked for typos and matched to the species information backbone of WorldsRegister of Marine Species (WoRMS); dichotomous keys and LSID were assigned to each taxon as scientificNameID. Eventdate and time were converted into ISO 8601 and verified with the field reports.

### Step description

"Swimmers" were manually isolated from passive sinking particles, picking them out from sediment trap samples under a ZEISS Discovery V.8 stereomicroscope at the National Research Council, Institute of Polar Sciences (CNR-ISP) section of Bologna (Italy) according to the protocol of Chiarini et al. (2013).

Samples were initially preserved at +4°C in a buffered 5% formaldehyde solution at the CNR-ISP laboratories. They were subsequently transferred to 96% ethanol and delivered to the MNA (Genoa section, Italy), where the previously unsorted “swimmers” were sorted, identified and counted. Some specimens were mounted on glass slides for study. All vouchers are now part of the biological collection of the MNA (Fig. 2), where a total of 905 vouchers have been assigned.

The "swimmer" taxa were identified to the lowest possible taxonomic level (Boltovskoy et al. 2003, Boxshall and Halsey 2004, Razouls et al. 2008, Castellani and Edwards 2017, Bonello et al. 2020). The term undefined indicates that the organism could not be taxonomically identified.

The online portal WoRMS was used to confirm acceptance of species names. When identification was inconclusive, only genus or family names were assigned (Fig. 2).

## Geographic coverage

### Description

Samples were collected in the Joides Basin, located in the central-north sector of the Ross Sea sector (Antarctica). Mooring positions: 74.0266°S Latitude and 175.0552°E Longitude (PNRA_1995-1996); 74.1744°S Latitude and 175.108°E Longitude (PNRA_2004-2005); 74.2533°S Latitude and 175.1086°E Longitude (PNRA_2014-2016).

### Coordinates

-75 and -74.5 Latitude; 163.5 and 165 Longitude.

## Taxonomic coverage

### Description

The "swimmer" diversity in the dataset is represented by a total of 210,411 individuals (comprising vials with single species isolated from bulk samples and glass slides with dissected or whole specimens) collected during three different Italian Antarctic expedition (i.e. when Mooring B has been under maintenance and sediment trap cups have been changed). A total of 906 MNA vouchers, with the highest proportions observed in PNRA_X (n = 393, 43.42%) and PNRA_XXIX (n = 357, 39.45%), followed by PNRA_XIX (n = 155, 17.13%). Swimmer species sampled using automatic sediment trap consist of two kingdoms, 11 phyla, 17 classes, 26 orders, 53 families, 68 genus and 68 species with six morphotypes not being identified (e.g., sp., spp., cf.), plus several undefined organisms that could not be classified at any taxonomic level.

The most abundant class is Copepoda, accounting for 43.54% of the total. This is followed by Polychaeta (17.13%), Hydrozoa (10.17%), Malacostraca (8.29%) and Gastropoda (6.85%). Other moderately represented groups include Ostracoda (5.75%) and Ophiuroidea (2.87%). The remaining classes show values below 1.5%, such as Thecostraca (1.33%), undefined (0.88%), Appendicularia and Asteroidea (both 0.77%), Cestoda (0.66%), Sagittoidea (0.55%) and other classes with even lower frequencies, such as Teleostei, Oligotrichea, Holothuroidea and Clitellata, each at 0.11% (Fig. 3).

At the PNRA_X time series, Copepoda (44.54%) and Polychaeta (20.10%) are the dominant classes, followed by Hydrozoa (9.16%), Malacostraca (7.63%), Gastropoda (5.34%) and Ostracoda (6.11%). Other classes show lower values: Ophiuroidea (3.56%), Asteroidea (1.02%), Cestoda (1.02%), Thecostraca (0.76%), Appendicularia (0.51%) and undefined (0.25%) (Fig. 4).

At the PNRA_XIX time series, Copepoda (40.00%) is once again the predominant class, followed by Malacostraca (12.26%), Gastropoda (11.61%), Polychaeta (9.03%), Ostracoda (8.37%) and Ophiuroidea (5.81%). These are followed by Thecostraca (4.52%), undefined (3.23%) and Appendicularia (1.94%). With lower percentages, Hydrozoa and Cestoda are both at 1.29% and Holothuroidea is at 0.65%.

At the PNRA_XXIX time series, Copepoda continues to dominate (43.98%), followed by Polychaeta (17.37%) and Hydrozoa (15.13%). In descending order, these are followed by Malacostraca (7.28%), Astropoda (6.44%), Ostracoda (4.20%), Sagittoidea (1.40%), Asteroidea (0.84%), Ophiuroidea (0.84%) and Appendicularia, Thecostraca and undefined (each at 0.56%). Finally, with the lowest percentage (0.28%), are Clitellata, Oligotrichea and Teleostei.

In general, Calanoida (36.69%) is the most abundant order (Fig. 5), followed by Phyllodocida (12.38%), undefined (9.06%), Cyclopoida (6.52%) and Pteropoda (5.97%). Other notable orders include Halocyprida (5.41%), Amphipoda (4.42%), Siphonophorae (3.87%), Narcomedusae (2.87%), Isopoda and Spionida (both at 2.43%). These are followed by orders with percentages below 2%, including Anthoathecata and Euphausiacea (both at 1.22%), Ophiacanthida (1.88%), Littorinimorpha (0.88%), Copelata (0.77%), Aphragmophora (0.55%), Terebellida (0.33%), Amphinomida (0.22%), Ophiurida (0.22%), Choreotrichida, Cumacea, Decapoda, Leptothecata, Podocopida and Scolecida (0.11%).

At PNRA_X time series, Calanoida (36.90%) is the most abundant order, followed by Phyllodocida (15.01%), undefined (8.40%), Cyclopoida (6.87%), Halocyprida (5.85%), Pteropoda (5.34%), Siphonophorae (4.33%), Amphipoda (4.07%) and Narcomedusae (3.05%). Other orders present at lower values include Spionida and Isopoda (2.80%), Ophiacanthida (1.79%), Terebellida (0.76%), Anthoathecata, Euphausiacea and Copelata (0.51%), Decapoda and Leptothecata (each at 0.25%) (Fig. 6).

At PNRA_XIX time series, Calanoida (32.90%) is the most represented order, followed by undefined (9.68%), Pteropoda (9.03%), Phyllodocida (8.39%), Halocyprida (7.72%), Cyclopoida (7.10%) and Amphipoda (6.45%). Ophiacanthida also shows a relevant presence (5.81%). Orders with lower percentages include Littorinimorpha, Isopoda and Euphausiacea (all at 2.58%), Copelata (1.94%) and Siphonophorae (1.29%). Finally, with marginal presence (0.65%), are the orders Cumacea, Podocopida and Scolecida.

At PNRA_XXIX time series, a clear dominance of Calanoida (38.12%) is observed, followed by Phyllodocida (11.20%) and undefined (9.52%). Other significantly represented orders include Cyclopoida (5.88%), Pteropoda (5.32%) and Siphonophorae (4.48%). Present, at lower percentages, are also Amphipoda, Halocyprida and Narcomedusae (all at 3.92%), Spionida (3.08%), Anthoathecata (2.52%), Isopoda (1.96%), Euphausiacea and Aphragmophora (both at 1.40%), Littorinimorpha (1.12%), Copelata, Ophiurida and Amphinomida (all at 0.56%) and Choreotrichida and Ophiacanthida (both at 0.28%).

Regarding the life stages range in the samples, in general, the dataset is composed by a majority of adults (73.48%), followed by copepodites stages (CII, CIII, CIV, CV and copepodids: 9.97%), nauplius (0.99%), cypris (1.10%), metazoan juveniles and larvae (juveniles, larvae and post-larvae 4.09%). Trochophore (0.22%), calyptopis (0.22%), decapodid (0.11%), praniza (0.11%), praniza III (0.33%), zuphea (1.10%), nectochaete (2.54%), auricolaria (0.11%), brachiolaria (0.22%), bipinnaria (0.55%), actinula (0.55%), trypanorhynch larvae (0.44%) and fragments of siphonophoran colony (3.87%) (Figs 7, 8).

The swimmers found inside the samples can be assigned to the following ecological category: holoplankton (75.36%), meroplankton (17.57%), benthos (5.97%), nekton (0.44%), while the remaining 0.66% could not be assessed and are reported as unidentified (Fig. 9).

### Taxa included

**Table:** 

Rank	Scientific Name	
kingdom	Animalia	
kingdom	Chromista	
phylum	Annelida	
phylum	Arthropoda	
phylum	Chaetognatha	
phylum	Chordata	
phylum	Ciliophora	
phylum	Cnidaria	
phylum	Echinodermata	
phylum	Heliozoa	
phylum	Mollusca	
phylum	Platyhelminthes	
class	Appendicularia	
class	Asteroidea	
class	Cestoda	
class	Clitellata	
class	Copepoda	
class	Gastropoda	
class	Holothuroidea	
class	Hydrozoa	
class	Malacostraca	
class	Oligotrichea	
class	Ophiuroidea	
class	Ostracoda	
class	Polychaeta	
class	Sagittoidea	
class	Teleostei	
class	Thecostraca	
order	Amphinomida	
order	Amphipoda	
order	Anthoathecata	
order	Aphragmophora	
order	Calanoida	
order	Choreotrichida	
order	Copelata	
order	Cumacea	
order	Cyclopoida	
order	Decapoda	
order	Euphausiacea	
order	Halocyprida	
order	Isopoda	
order	Leptothecata	
order	Littorinimorpha	
order	Narcomedusae	
order	Ophiacanthida	
order	Ophiurida	
order	Phyllodocida	
order	Podocopida	
order	Pteropoda	
order	Scolecida	
order	Siphonophorae	
order	Spionida	
order	Terebellida	
family	Aetideidae	
family	Agalmatidae	
family	Augaptilidae	
family	Bougainvilliidae	
family	Calanidae	
family	Capulidae	
family	Centropagidae	
family	Cirolanidae	
family	Cirratulidae	
family	Clausocalanidae	
family	Cliidae	
family	Clionidae	
family	Corophiidae	
family	Cymothoidae	
family	Diphyidae	
family	Epimeriidae	
family	Euchaetidae	
family	Euphausiidae	
family	Euphausiidae	
family	Glyceridae	
family	Gnathiidae	
family	Halocyprididae	
family	Halocyprididae	
family	Hyperiidae	
family	Iospilidae	
family	Limacinidae	
family	Limacinidae	
family	Lopadorrhynchidae	
family	Metridinidae	
family	Mitrocomidae	
family	Nannastacidae	
family	Nematocarcinidae	
family	Nereididae	
family	Oikopleuridae	
family	Oithonidae	
family	Oncaeidae	
family	Ophiacanthidae	
family	Ophiuridae	
family	Phaennidae	
family	Phyllodocidae	
family	Polynoidae	
family	Pontocyprididae	
family	Pyrostephidae	
family	Pyrostephidae	
family	Resomiidae	
family	Rhincalanidae	
family	Sagittidae	
family	Scalibregmatidae	
family	Scolecitrichidae	
family	Solmundaeginidae	
family	Spinocalanidae	
family	Spionidae	
family	Tharybidae	
family	Tomopteridae	
family	Tryphosidae	
family	Tubulariidae	
genus	*Aetideopsis Sars* G.O., 1903	
genus	*Agalma* Eschscholtz, 1825	
genus	*Alacia* Poulsen, 1973	
genus	*Aphelochaeta* Blake, 1991	
genus	*Archiconchoecemma* Chavtur & Stovbun, 2003	
genus	*Bargmannia* Totton, 1954	
genus	*Boroecia* Poulsen, 1973	
genus	*Bougainvillia* Lesson, 1830	
genus	*Caecognathia* Dollfus, 1901	
genus	*Calanoides* Brady, 1883	
genus	*Calanus* Leach, 1816	
genus	*Campylaspis* G.O. Sars, 1865	
genus	*Centropages* Krøyer, 1849	
genus	*Chiridiella* Sars G.O., 1907	
genus	*Chirundina* Giesbrecht, 1895	
genus	*Clio* Linnaeus, 1767	
genus	*Clione* Pallas, 1774	
genus	*Conchoecia* Dana, 1849	
genus	*Cryocapulus* Schiaparelli, Bouchet, Fassio & Oliverio, 2020	
genus	*Ctenocalanus* Giesbrecht, 1888	
genus	*Ectopleura* L. Agassiz, 1862	
genus	*Epimeria* A. Costa in Hope, 1851	
genus	*Euchirella* Giesbrecht, 1888	
genus	*Euphausia* Dana, 1850	
genus	*Gaetanus* Giesbrecht, 1888	
genus	*Haloptilus* Giesbrecht, 1898	
genus	*Haplocheira* Haswell, 1879	
genus	*Harmothoe* Kinberg, 1856	
genus	*Heteroptilus* Sars G.O., 1920	
genus	*Iospilus* Viguier, 1886	
genus	*Limacina* Bosc, 1817	
genus	*Lopadorrhynchus* Grube, 1855	
genus	*Metaconchoecia* Howe, 1955	
genus	*Metridia* Boeck, 1865	
genus	*Microcalanus* Sars G.O., 1903	
genus	*Mitrocomella* Haeckel, 1879	
genus	*Neoscolecithrix* Canu, 1896	
genus	*Nereis* Linnaeus, 1758	
genus	*Oikopleura* Mertens, 1830	
genus	*Oithona* Baird, 1843	
genus	*Oncaea* Philippi, 1843	
genus	*Ophiacantha* Müller & Troschel, 1842	
genus	*Ophiura* Lamarck, 1801	
genus	*Orchomenella* Sars, 1890	
genus	*Paraeuchaeta* Scott A., 1909	
genus	*Paramollicia* Poulsen, 1973	
genus	*Pelagobia* Greeff, 1879	
genus	*Phalacrophorus* Greeff, 1879	
genus	*Polydora* Bosc, 1802	
genus	*Pontocypria* Müller, 1894	
genus	*Pontoptilus* Sars G.O., 1905	
genus	*Pseudochirella* Sars G.O., 1920	
genus	*Resomia* Pugh, 2006	
genus	*Rhincalanus* Dana, 1852-1853	
genus	*Rhynchonereella* Costa, 1864	
genus	*Sagitta* Quoy & Gaimard, 1827	
genus	*Scaphocalanus* Sars G.O., 1900	
genus	*Sclerocheilus* Grube, 1863	
genus	*Solmundella* Haeckel, 1879	
genus	*Spinocalanus* Giesbrecht, 1888	
genus	*Tomopteris* Eschscholtz, 1825	
genus	*Torrea* Quatrefages, 1850	
genus	*Triconia* Böttger-Schnack, 1999	
genus	*Undinella* Sars G.O., 1900	
genus	*Vanadis* Claparède, 1870	
genus	*Xanthocalanus* Giesbrecht, 1893	
species	*Aetideopsis antarctica* (Wolfenden, 1908)	
species	*Alacia belgicae* (Müller, 1906)	
species	*Alacia hettacra* (Muller, 1906)	
species	*Aphelochaeta cincinnata* (Ehlers, 1908)	
species	*Aphelochaeta marioni* (Saint-Joseph, 1894)	
species	*Bargmannia elongata* Totton, 1954	
species	*Boroecia antipoda* (Müller, G. W., 1906)	
species	*Bougainvillia macloviana* Lesson, 1830	
species	*Calanoides acutus* (Giesbrecht, 1902)	
species	*Calanoides carinatus* (Krøyer, 1849)	
species	*Calanus propinquus* Brady, 1883	
species	*Calanus simillimus* Giesbrecht, 1902	
species	*Campylaspis heterotuberculata* Muradian, 1976	
species	*Centropages brachiatus* (Dana, 1849)	
species	*Chirundina stretsii* Giesbrecht, 1895	
species	*Clio pyramidata* Linnaeus, 1767	
species	*Clione limacina* Phipps, 1774	
species	*Cochoecia magna* Claus, 1874	
species	*Cryocapulus subcompressus* (Pelseneer, 1903)	
species	*Ctenocalanus citer* Heron & Bowman, 1971	
species	*Ctenocalanus vanus* Giesbrecht, 1888	
species	*Euchirella rostrata* (Claus, 1866)	
species	*Euphausia superba* Dana, 1850	
species	*Gaidius tenuispinus* (Sars G.O., 1900)	
species	*Haloptilus oxycephalus* (Giesbrecht, 1889)	
species	*Harmothoe crostensis* (McIntosh, 1885)	
species	*Iospilus phalacoides* Viguier, 1886	
species	*Limacina rangii* (A. d'Orbigny, 1835)	
species	*Limacina retroversa* (J. Fleming, 1823)	
species	*Metaconchoecia australis* (Gooday, 1981)	
species	*Metridia gerlachei* Giesbrecht, 1902	
species	*Metridia longa* (Lubbock, 1854)	
species	*Metridia lucens* Boeck, 1865	
species	*Microcalanus pigmaeus* (Sars G.O., 1900)	
species	*Oithona frigida* Giesbrecht, 1902	
species	*Oithona plumifera* Baird, 1843	
species	*Oithona similis* Claus, 1866	
species	*Oncaea curvata* Giesbrecht, 1902	
species	*Paraeuchaeta antarctica* (Giesbrecht, 1902)	
species	*Paraeuchaeta exigua* (Wolfenden, 1911)	
species	*Paramollicia major* Müller G. W., 1906	
species	*Pelagobia longicirrata* Greef R., 1879	
species	*Phalacrophorus pictus* Greeff, 1879	
species	*Polydora ciliata* Johnston, 1838	
species	*Pontocypria helenae* Maddocks, 1968	
species	*Pontoptilus pertenuis* Sars G.O., 1907	
species	*Pseudochirella hirsuta* (Wolfenden, 1905)	
species	*Pseudochirella spectabilis* (Sars G.O., 1900)	
species	*Resomia convoluta* (Moser, 1925)	
species	*Rhincalanus gigas* Brady, 1883	
species	*Rhincalanus nasutus* Giesbrecht, 1888	
species	*Rhynchonereella petersii* (Langerhans, 1880)	
species	*Scaphocalanus subbrevicornis* (Wolfenden, 1911)	
species	*Sclerocheilus antarticus* Ashworth, 1915	
species	*Solmundella bitentaculata* (Quoy & Gaimard, 1833)	
species	*Spinocalanus abyssalis* Giesbrecht, 1888	
species	*Tomopteris planktonis* Apstein, 1900	
species	*Torrea candida* (Delle Chiaje, 1841)	
species	*Triconia antarctica* (Heron, 1977)	
species	*Triconia conifera* (Giesbrecht, 1891)	
species	*Undinella simplex* (Wolfenden, 1906)	
species	*Vanadis longissima* (Levinsen, 1885)	

## Temporal coverage

**Data range:** 1994-12-15 – 1996-1-13; 2004-1-14 – 2006-1-01; 2014-1-01 – 2016-1-14.

## Collection data

### Collection name

Antarctic_”swimmers”_Mooring_B

### Collection identifier

https://scientific-collections.gbif.org/collection/a57a1dc1-706c-42db-bbad-1e68d9685439

### Parent collection identifier

Italian National Antarctic Museum (section of Genoa)

### Specimen preservation method

the methods used for the conservation of the species were: conservation of the specimens in jars in ethanol 96%, slides with whole or dissected organisms (fixed in glycerol) and frozen at -20°C.

## Usage licence

### Usage licence

Other

### IP rights notes

The dataset was published under the licence CC-BY 4.0.

## Data resources

### Data package title

Checklist of organisms (“swimmers”) collected by Sediment Trap on Mooring B (Ross Sea shelf, Joides Basin, Antarctica) in years 1995-1996, 2004-2005 and 2014-2016 

### Resource link

https://ipt.biodiversity.aq/archive.do?r=mnait_sediment_trap_mooring_b&v=1.3

### Alternative identifiers

https://ipt.biodiversity.aq/resource?r=mnait_sediment_trap_mooring_b

### Number of data sets

1

### Data set 1.

#### Data set name

Swimmers collected by an automatic sediment trap on Mooring B (Ross Sea, Antarctica)

#### Data format

Darwin Core

#### Description

This dataset is built on information from the “swimmers” specimens analysed in this work (Grillo et al. 2026). The samples were pooled into a single dataset. This dataset will be useful to investigate the community structure of “swimmers” and their relative larval stages.

**Data set 1.** 

Column label	Column description
occurrenceID	A global unique identifier for the Occurrence (as opposed to a particular digital record of the occurrence).
institutionCode	The name (or acronym) in use by the institution (as showed on the Global Registry of Scientific Collections) having custody of the object(s) or information referred to in the record.
instituitonID	An identifier for the institution having custody of the object(s) or information referred to in the record.
collectionCode	The name, acronym, code or initialism identifying the collection or dataset from which the record was derived (as showed on the Global Registry of Scientific Collections).
collectionID	The name, acronym, coden or initialism identifying the collection or dataset from which the record was derived (as showed on the Global Registry of Scientific Collections).
catalogNumber	An identifier of any form assigned by the source within a physical collection or digital dataset for the record which may not be unique, but should be fairly unique in combination with the institution and collection code.
basisOfRecord	The specific nature of the data record and is here always reported as “PreservedSpecimen”.
Type	Defines the nature of the resource, here is always “PhysicalObject”.
scientificName	The identification at the lowest taxonomic rank, without authorship information.
TaxonRank	The taxonomic rank of the most specific name in the scientificName.
Kingdom	The full scientific name of the kingdom in which the taxon is classified.
Phylum	The full scientific name of the phylum in which the taxon is classified.
Class	The full scientific name of the class in which the taxon is classified.
Order	The full scientific name of the order in which the taxon is classified.
Family	The full scientific name of the family in which the taxon is classified.
Genus	The full scientific name of the genus in which the taxon is classified.
specificEpithet	The name of the first or species epithet of the scientificName.
scientificNameAuthorship	The authorship information for the scientificName formatted according to the conventions of the applicable nomenclaturalCode.
identificationQualifier	A brief phrase or a standard term (sp., spp.) to express the determiner's doubts about the Identification.
ScientificNameID	Marinespecies.org taxon number.
individualCount	The type of quantification system used, such as the number of individuals.
Sex	The sex, male or female, of the identified specimens.
lifeStage	The life stage of organisms. In detail: Actinula, Adult or other: Adult or another larval stage, Auricolaria, Bipinnaria, Brachiolaria, Calyptopis, CII: copepodite II, CIII: copepodite III, CIV: copepodite IV; CV: copepodid: Copepodid not identified, Cypris, Decapodid, Juvenile, Larvae, Nauplius, Nectochaete, Piece of colony, Post-larva, Praniza, Praniza 3: third larval stage of ispoda, trochophore, Trypanorhynch larvae, Zuphea.
occurrenceRemarks	Italian Antarctic campaign in which the organisms were sampled.
eventDate	Date the organism were sampled.
decimalLatitude	The geographic latitude (in decimal degrees, using the spatial reference system given in geodeticDatum).
decimalLongitude	The geographic latitude (in decimal degrees, using the spatial reference system given in geodeticDatum).
geodedicDatum	Spatial reference system (WGS84) upon which the geographic coordinates given in decimalLatitude and decimalLongitude are based.
coordinateUncertaintyInMetres	The horizontal distance in metres, calculated from the provided decimalLatitude and decimalLongitude, representing the radius of the smallest circle that fully encloses the location.
coordinatePrecision	A decimal value that specifies how precise the decimalLatitude and decimalLongitude coordinates are.
MinimumDepthInMetres	Minimum sampling depth during event in metres.
MaximumDepthInMetres	Maximum sampling depth during event in metres.
samplingProtocol	Gear used to collect specimens and relative DOI of manuscript in which the sampling method is described.
eventRemarks	Sample number of the sediment trap.
Preparations	A list of preparations and preservation methods for a specimen. In detail: whole organism (96% ethanol), whole organism (slide fixed in glycerol) and dissected organism (slide fixed in glycerol).
taxonRemarks	Remarks on taxa, in this case, in which ecological category the analysed species occupy.
samplingEffort	Information on mouth sediment trap size (0.5 m).
occurrenceStatus	A statement about the presence or absence of a specimen.
Continent	Continent where the organisms were sampled.
countryCode	The standard code for the country where the organisms were sampled.
recordedBy	Surname and name of the personnel who collected the samples.
recordedByID	ORCID of the personnel who collected the samples.
identifiedBy	Name and surname of the personnel who analysed and recognised the single species.
identifiedByID	ORCID of the personnel who analysed and recognised the single species.
eventID	Unique code with data relating to the campaign and sampling date.
verbatimTaxonRank	The most specific taxonomic rank given in the original scientific name (e.g. sp., spp.).

## Figures and Tables

**Figure 1.**
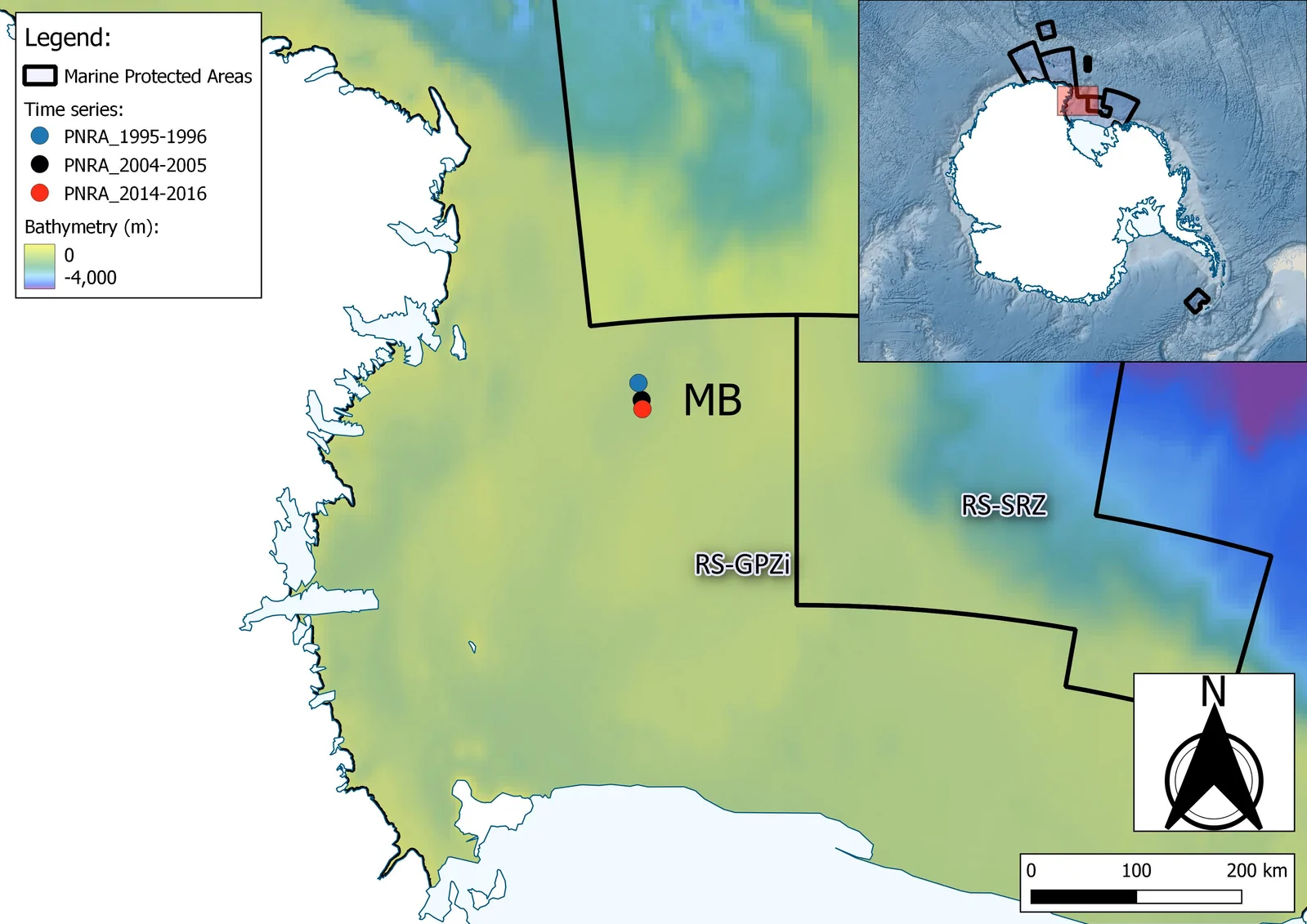
Mooring B (MB) position and study area. The map shows the three time series included in the dataset in the Joides Basin (Ross sea, Antarctica). This map was produced using the Quantarctica package (Matsuoka et al. 2021) and QGIS programme (QGIS Development Team 2023). Acronym: Ross Sea General Protection Zone section I (RS-GPZi) and Ross Sea Special Research Zone (RS-SRZ).

**Figure 2.**
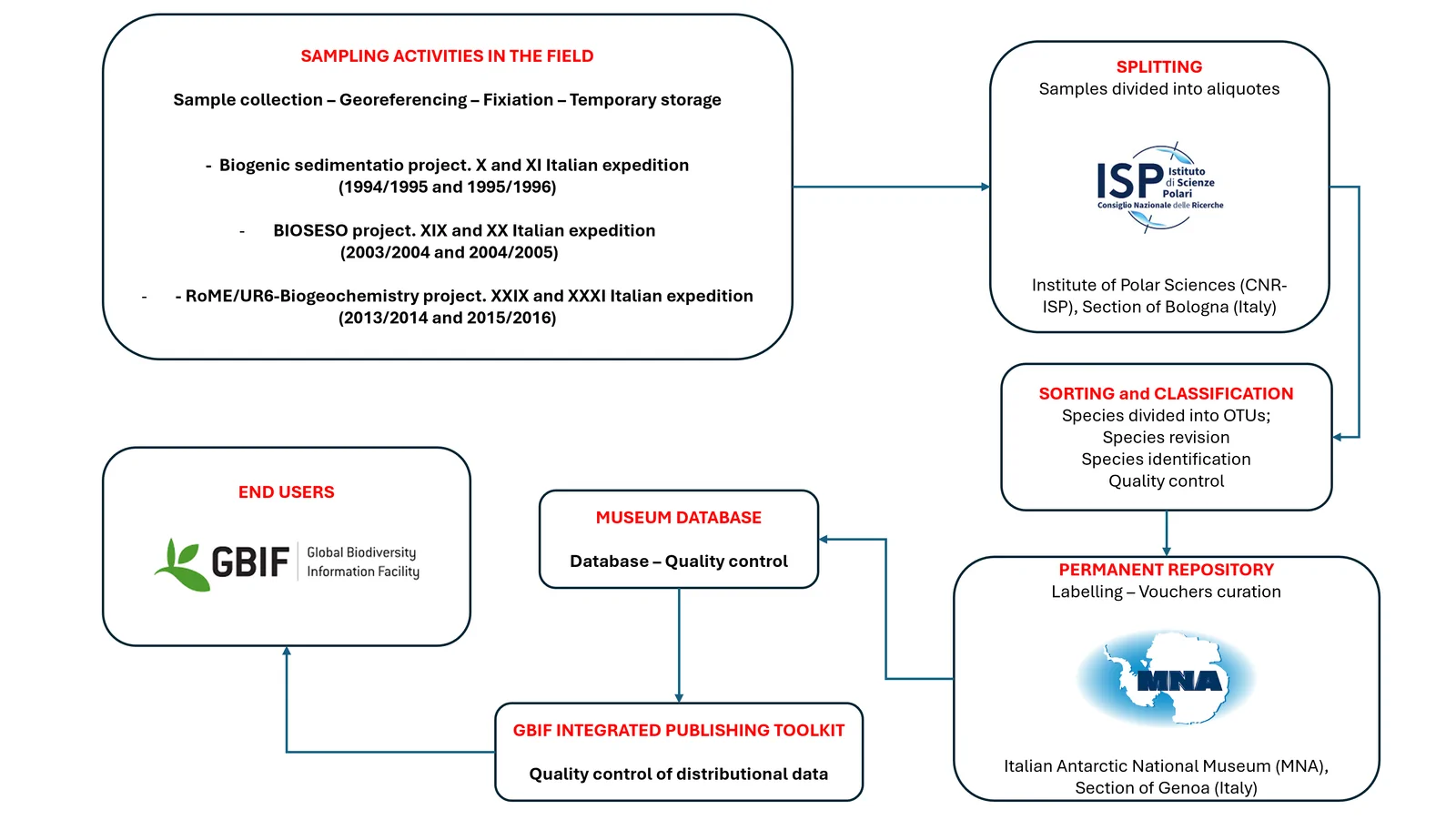
Flowchart illustrating the complete process of dataset creation and publication. The diagram summarises the sampling, sorting (which includes the Operational Taxonomic Units (OTUs) classification, based on morphological characters) and taxonomic revision operations, ending with the management of vouchers and the publication of data via GBIF.

**Figure 3.**
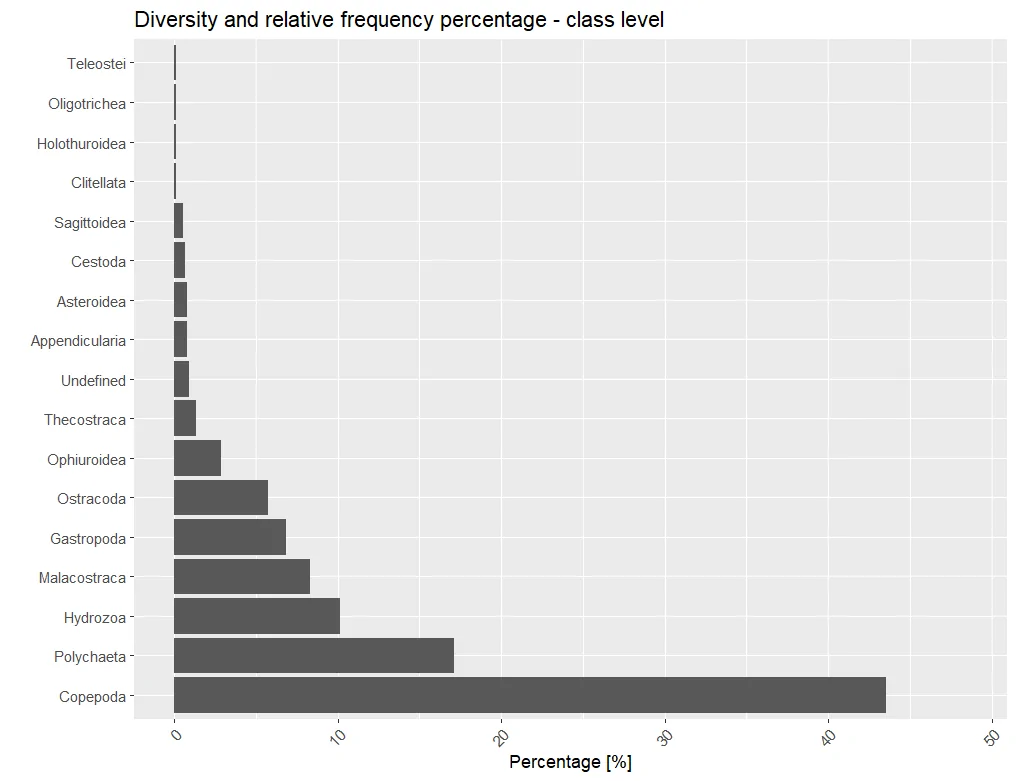
Class-level diversity and relative frequency (%), based on the number of individuals in the dataset.

**Figure 4.**
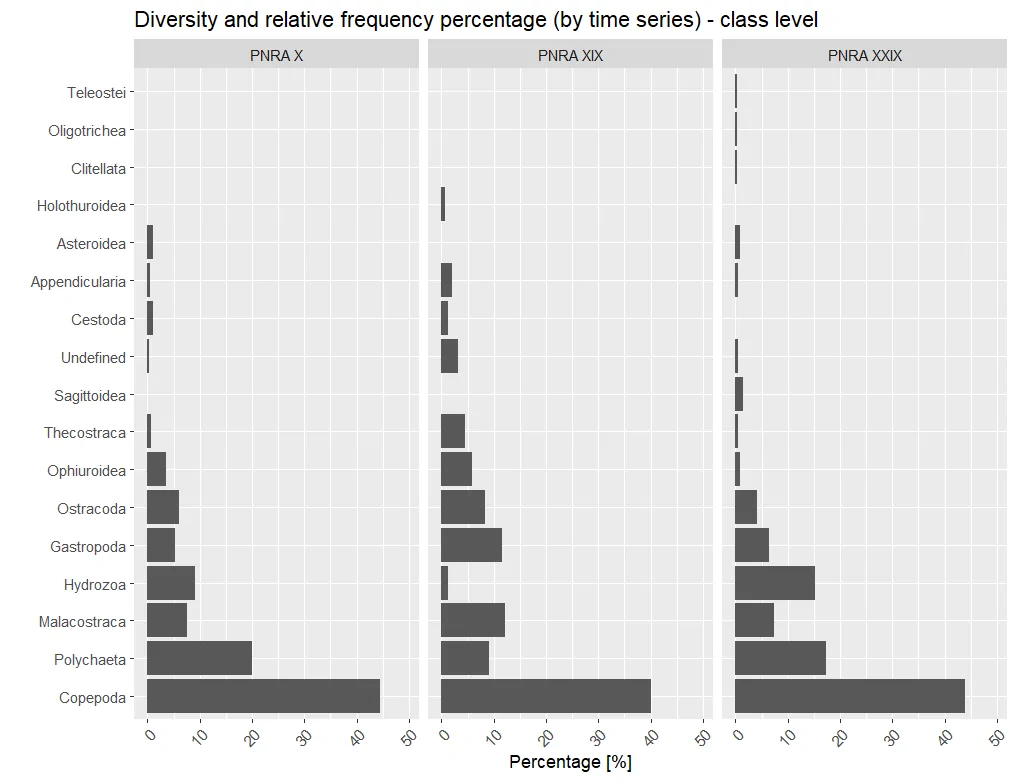
Class-level diversity and relative frequency (%) of individuals in the dataset, analysed by time series.

**Figure 5.**
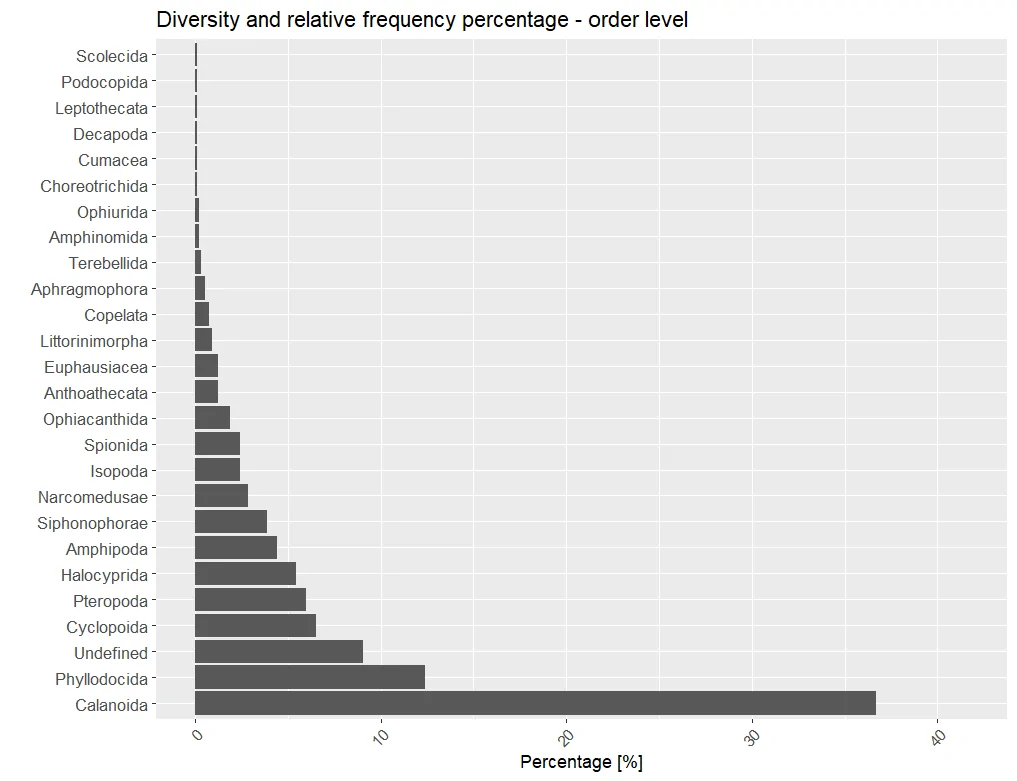
Diversity and relative frequency (%) at the order level for the number of individuals in the dataset.

**Figure 6.**
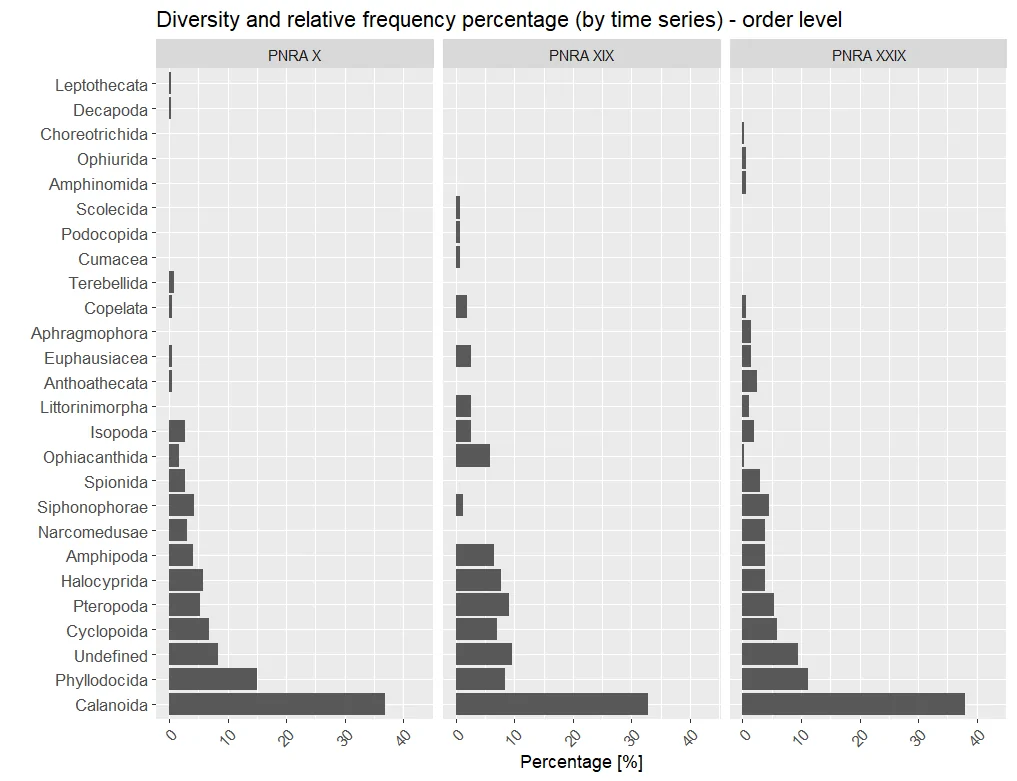
Order-level diversity and relative frequency (%) of individuals in the dataset, analysed by time series.

**Figure 7.**
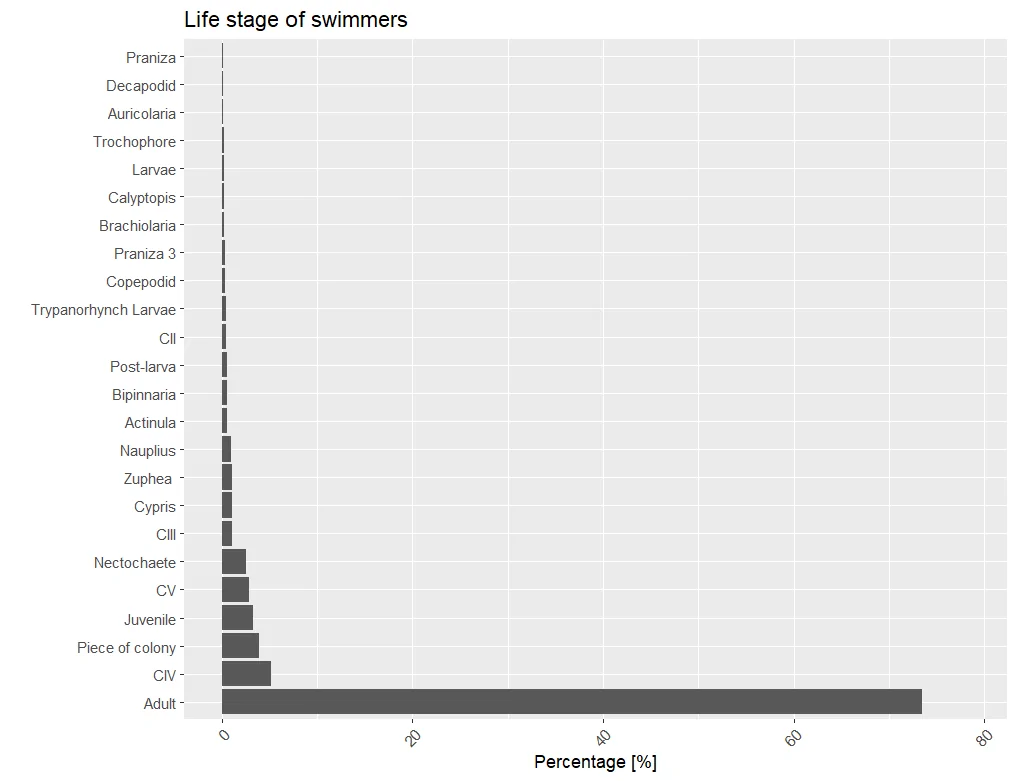
Diversity and relative frequency (%) at the class level for the number of life stages in the dataset.

**Figure 8.**
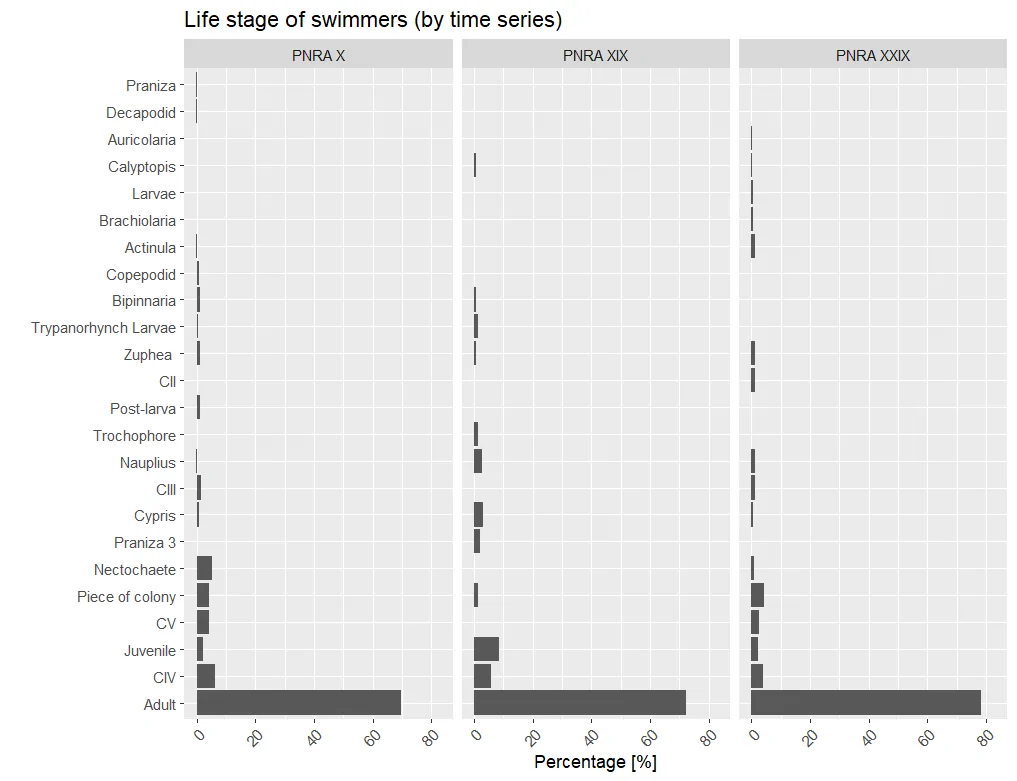
Diversity and relative frequency (%) of life stage in the dataset, analysed by time series.

**Figure 9.**
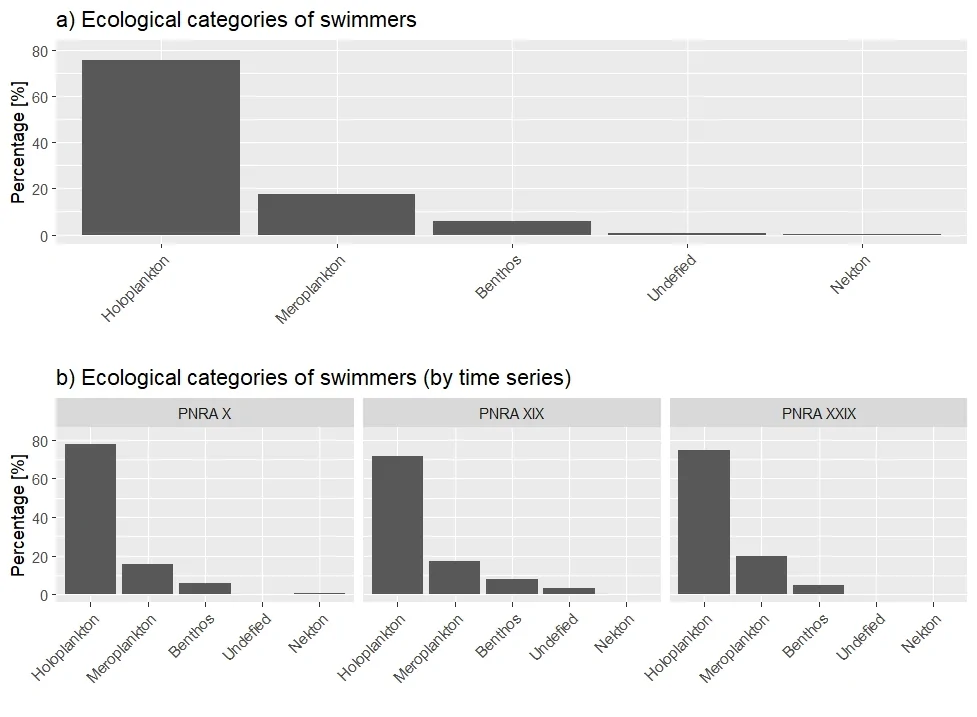
Diversity and relative frequency (%). **a** Diversity and relative frequency percentages at the life stage level found in the dataset; **b** Diversity and relative frequency percentages at the life stage level in the dataset, analysed across time series.
